# Atorvastatin Induces Mitochondria-Dependent Ferroptosis *via* the Modulation of Nrf2-xCT/GPx4 Axis

**DOI:** 10.3389/fcell.2022.806081

**Published:** 2022-03-03

**Authors:** Qi Zhang, Hang Qu, Yinghui Chen, Xueyang Luo, Chong Chen, Bing Xiao, Xiaowei Ding, Pengjun Zhao, Yanan Lu, Alex F. Chen, Yu Yu

**Affiliations:** ^1^ Institute for Developmental and Regenerative Cardiovascular Medicine, Xinhua Hospital, School of Medicine, Shanghai Jiao Tong University, Shanghai, China; ^2^ Department of Pediatric Cardiology, Xinhua Hospital, School of Medicine, Shanghai Jiao Tong University, Shanghai, China

**Keywords:** ferroptosis, atorvastatin, cardiomyocytes, lipid peroxidation, ROS

## Abstract

As one of the cornerstones of clinical cardiovascular disease treatment, statins have an extensive range of applications. However, statins commonly used have side reactions, especially muscle-related symptoms (SAMS), such as muscle weakness, pain, cramps, and severe condition of rhabdomyolysis. This undesirable muscular effect is one of the chief reasons for statin non-adherence and/or discontinuation, contributing to adverse cardiovascular outcomes. Moreover, the underlying mechanism of muscle cell damage is still unclear. Here, we discovered that ferroptosis, a programmed iron-dependent cell death, serves as a mechanism in statin-induced myopathy. Among four candidates including atorvastatin, lovastatin, rosuvastatin, and pravastatin, only atorvastatin could lead to ferroptosis in human cardiomyocytes (HCM) and murine skeletal muscle cells (C2C12), instead of human umbilical vein endothelial cell (HUVEC). Atorvastatin inhibits HCM and C2C12 cell viability in a dose-dependent manner, accompanying with significant augmentation in intracellular iron ions, reactive oxygen species (ROS), and lipid peroxidation. A noteworthy investigation found that those alterations particularly occurred in mitochondria and resulted in mitochondrial dysfunction. Biomarkers of myocardial injury increase significantly during atorvastatin intervention. However, all of the aforementioned enhancement could be restrained by ferroptosis inhibitors. Mechanistically, GSH depletion and the decrease in nuclear factor erythroid 2-related factor 2 (Nrf2), glutathione peroxidase 4 (GPx4), and xCT cystine–glutamate antiporter (the main component is SLC7A11) are involved in atorvastatin-induced muscular cell ferroptosis and damage. The downregulation of GPx4 in mitochondria-mediated ferroptosis signaling may be the core of it. In conclusion, our findings explore an innovative underlying pathophysiological mechanism of atorvastatin-induced myopathy and highlight that targeting ferroptosis serves as a protective strategy for clinical application.

## Introduction

Statins, inhibitors of 3-hydroxy-3-methyl-glutaryl-coenzyme A (HMG-CoA) reductase, are the most extensively applied cholesterol-reducing medicine to prevent cardiovascular events (Downs et al., 1998). In addition, statins exhibit pleiotropic cellular effects and have potential role in the treatment of many other conditions, including peripheral arterial disease, idiopathic dilated cardiomyopathy, ventricular arrhythmias, and cancer. Although statins are generally safe, treatment adherence is not optimal in a considerable proportion of patients, resulting in poor cardiovascular outcomes. Among them, the adverse effects of statin-associated muscle symptoms (SAMS) have the greatest health burden (Thompson et al., 2016; Bergt et al., 2017; Ward et al., 2019), including muscular pain, nocturnal muscle cramping, weakness, rare rhabdomyolysis, and amyotrophic lateral sclerosis (ALS)-like muscle wasting conditions (Bai et al., 2021). To date, there is no therapeutic approach for eliminating the adverse effects of statins, and the molecular mechanism underlying the etiology and pathophysiology of the statin-relative adverse effects still remain largely unknown.

Previous studies about SAMS focused on skeletal muscle, and the majority of mechanisms were centered on mitochondrial dysfunction, oxidative stress, immune-mediated necrotizing myopathy, and inhibition of isoprenylation of small G-proteins, owing to impaired mevalonate metabolism upon skeletal muscle damage (Cao et al., 2009; Musset et al., 2016; Thompson et al., 2016; Tiniakou and Christopher-Stine, 2017). But these just account for only a small percentage of it. As for cardiac muscle, a special kind of striated muscle, previous reports demonstrated that the mechanisms of SAMS in cardiac muscle mainly concentrate on mitochondrial dysfunction and apoptosis (Bonifacio et al., 2016; Godoy et al., 2019).

Programmed cell death is a basic physiological process in aging, development, and tissue homeostasis, which is often dysregulated in different pathological conditions. As such, a newly recognized form of programmed cell death, which is termed as ferroptosis (Dixon et al., 2012), has been implicated to play a critical pathogenic role in diverse cardiovascular conditions such as drug side effect or ischemia/reperfusion injury (Tadokoro et al., 2020; Menon et al., 2021). Those reports highlight that targeting ferroptosis may be a cardioprotective strategy (Fang et al., 2019; Tadokoro et al., 2020; Menon et al., 2021). There are two cellular protective systems against ferroptosis: the glutathione-dependent pathway involving GPx4 as well as System Xc−, and the CoQ-dependent pathway involving FSP1 on the plasma membrane (Bersuker et al., 2019; Doll et al., 2019) as well as DHODH in mitochondria (Mao et al., 2021). Whether ferroptosis was involved in the adverse effects of statins and the possible mechanism remains unclear.

In this study, we found that ferroptosis is responsible for atorvastatin-induced muscular injury both in cardiomyocytes and skeletal muscle cells. Mechanistically, atorvastatin induces ferroptosis by suppressing the intracellular anti-oxidative system, Nrf2-xCT/GPx4 pathway, resulting in the lethal lipid peroxidation within muscular cells. Our results for the first time demonstrated that ferroptosis is the pathogenic mechanism of atorvastatin-induced myopathy, providing a novel therapeutic target for improving atorvastatin application strategies.

## Materials and Methods

### Cell Culture and Drug Treatments

HCM, C2C12 and HUVEC were cultured in high-glucose DMEM (Gibco BRL, USA) supplemented with 5% fetal bovine serum (Gibco BRL, USA) without antibiotics in acceptable conditions (5% CO_2_, 37°C). Ferrostatin-1 (Fer-1), deferoxamine mesylate (DFO), liproxstatin-1 (Lip-1), RSL3, Z-VAD-FMK and mitoTEMPO (MT) were purchased from Selleck (Selleck, China). Atorvastatin, lovastatin, pravastatin, and rosuvastatin were obtained from MCE (MedChemExpress, China). Fer-1, DFO, Lip-1, RSL3, Z-VAD-FMK and MT were dissolved in dimethylsulfoxide (DMSO) and diluted in a culture medium at ultimate concentrations (0.5 μM with RSL3; 1 μM with Fer-1; 80 μM with DFO; 1 μM with Lip-1; 10 mM with mitoTEMPO; and 2 μM with Z-VAD-FMK). Statins were stocked as 50 mM solutions in DMSO. Final concentrations ranged from 10 to 160 μM.

### Cell Viability Assay

In brief, the cultured cells were seeded at the density of 5,000 cells per well in 96-well plates with three replicates. Cells were treated with statins at the dosages of 0, 10, 20, 40, 80 and 160 μM in experimental groups. Meanwhile, the normal culture medium without addition of any statins was set as the control group. Then a Cell Counting Kit-8 (CCK-8) (Sigma, USA) was used to assess cell viability in line with manufacturer’s instructions. Cell viability = Absorbance of (experimental group − blank control group)/absorbance of (control group − blank control group).

### Iron Assay

FerroOrange and Mito-FerroGreen probes (DojinDo, Japan) were employed to assess intracellular and mitochondrial iron content, respectively. HCM and C2C12 cells were seeded into 24-well plates and exposed to atorvastatin at the concentrations of 40 uM alone, or other treatments for 24 h. After that, the cells were stained with FerroOrange (1 μM) or Mito-FerroGreen (5 μM) probes along with Hoechst 33,342 (5 μg/ml). After 30 min of lucifuge incubation, the cells were then evaluated by a Leica SP8 confocal fluorescence microscope (Leica Microsystems, Germany).

### Detection of Cellular ROS Levels

Intracellular and mitochondrial ROS levels were detected using fluorescent measurement assay by the uptake of 1 μM DCFH-DA (Beyotime, China) and 5 μM MitoSOX Red (Thermo Scientific, USA), respectively. Images were obtained by a Leica SP8 confocal laser scanning microscope (Leica Microsystems, Germany). Fluorescence intensity was measured by ImageJ (National Institutes of Health, USA). The mean fluorescent intensity of each group was standardized to that of the control group.

### Lipid Peroxidation Levels

Lipid peroxidation was measured using C11-BODIPY581/591(Invitrogen™, USA) and MitoPeDPP (Dojindo, Japan) kits. After incubation with C11-BODIPY581/591 and MitoPeDPP solution, lipid peroxidation in the mitochondrial inner membrane was fluorometrically measured at 470 nm using a fluorescence microscope (Leica Microsystems, Germany). The investigation of MDA consistency was strictly conducted in accordance with the manufacturer’s instructions (Beyotime, China). For the MDA assay, thiobarbituric acid (TBA) was supplied to supernatants of cell homogenate and softly oscillated for the formation of TBA-MDA mixture. Then the mixture was detected spectrophotometrically at 535 nm, and the BCA protein determination kit (Beyotime, China) was employed for total protein quantification.

### Mitochondrial Membrane Potential Levels

Mitochondrial membrane potential (MMP) was estimated by TMRM (Thermo Fisher Scientific, USA) staining. HCM and C2C12 cells were stained with TMRM at an ultimate concentration of 200 nmol/L. Images were acquired by a fluorescence microscope (Leica Microsystems, Germany).

### MitoTracker Red CMXRos Staining

To locate the mitochondria, cultured cells were stained with 200 nM MitoTracker Red CMXRos (Thermo Fisher Scientific, USA) for 15 min at room temperature in the dark. Subsequently, cells were washed with PBS and then randomly captured by using a confocal microscope (Leica Microsystems, Germany).

### Transmission Electron Microscopy

Cells were fixed with 2.5% glutaraldehyde in 0.1 mol/L of Sorenson’s buffer (0.1 mol/L H2PO4 and 0.1 mol/L HPO4 [pH 7.2]) at 4°C overnight, washed three times with PBS, and then treated with 1% OsO4 in 0.1 mol/L of Sorenson’s buffer for 2 h at room temperature. After dehydration using an ethanol series and acetone (100%), cells were embedded in acetone and embed-812. Thin sections were cut using an ultramicrotome (Leica Microsystems, Germany), and the sections were subjected to double staining with uranyl acetate and lead citrate and then observed using a transmission electron microscope (Hitachi HT7800, Japan).

### Quantitative Real-Time PCR

Total RNA from C2C12 cells was extracted by using the EZ-press RNA purification kit (EZBioscience, USA), according to manufacturer’s instruction. Then RNA was reverse-transcribed to cDNA by using a reverse transcription kit (Takara, China). Quantitative PCR was performed by using SYBR Green master mix (Takara, China) on a real-time PCR system (Biorad, USA). The results were normalized to *β*-actin as an internal control. All data were expressed in terms of fold-change relative to the control samples.

### Enzyme-Linked Immunosorbent Assay

The level of CoQ10 and CKMB in HCM was measured by ELISA. After different treatment conditions, the supernatants of HCM were collected for ELISA using CoQ10 and CKMB Enzyme Immunoassay (EIA) kits (Clond-Clone, China), respectively.

### GSH/GSSG and GSH-Px Detection

The commercialized GSH/GSSG assay kit and the GSH-Px assay kit were used to measure the intracellular levels of GSH, GSSG, and GSH-Px, respectively (Beyotime, China), in accordance with the instructions of the manufacturer.

### Western Blot

Cells were lysed with a cold radio-immunoprecipitation assay (RIPA) (Beyotime, China) lysis buffer plus protease and a phosphatase inhibitor. The protein concentrations were detected by a bicinchoninic acid kit (BCA) (Beyotime, China). Protein sample were separated *via* 12% SDS-PAGE and moved to positively charged nylon (PVDF) membranes (Immobilon-P, Millipore, Switzerland). After blocking with bovine serum albumin (BSA), the membranes were detected using anti-Nrf2 (1:5,000, Abcam), anti-GPX4 (1:1,000, Abcam), anti-SLC7A11 (1:1,000, Abcam), anti-DHODH (1:1,000, Abcam) and anti-β-actin (1:10,000, Abclone). Horseradish peroxidase-conjugated secondary antibodies were diluted 1:10,000 and incubated for 1 h, and chemiluminescence solution and an electrochemiluminescence (ECL) kit (Millipore, USA) were used to detect protein band.

### Statistical Analysis

Data were exhibited as mean ± standard deviation (SD). Statistical analysis was performed using unpaired Student’s t-tests, one-way or two-way ANOVA, followed by Dunnett’s method for multiple comparisons, and Prism 8.0 software (GraphPad Software, United States) was used to perform all the statistical testing and the creation of graphs. Statistical significance was considered at *p* < 0.05. All the experiments were repeated at least three times.

## Results

### Ferroptosis Contributes to Atorvastatin-Induced Cell Damage in HCM and C2C12 Cells

**FIGURE 1:**
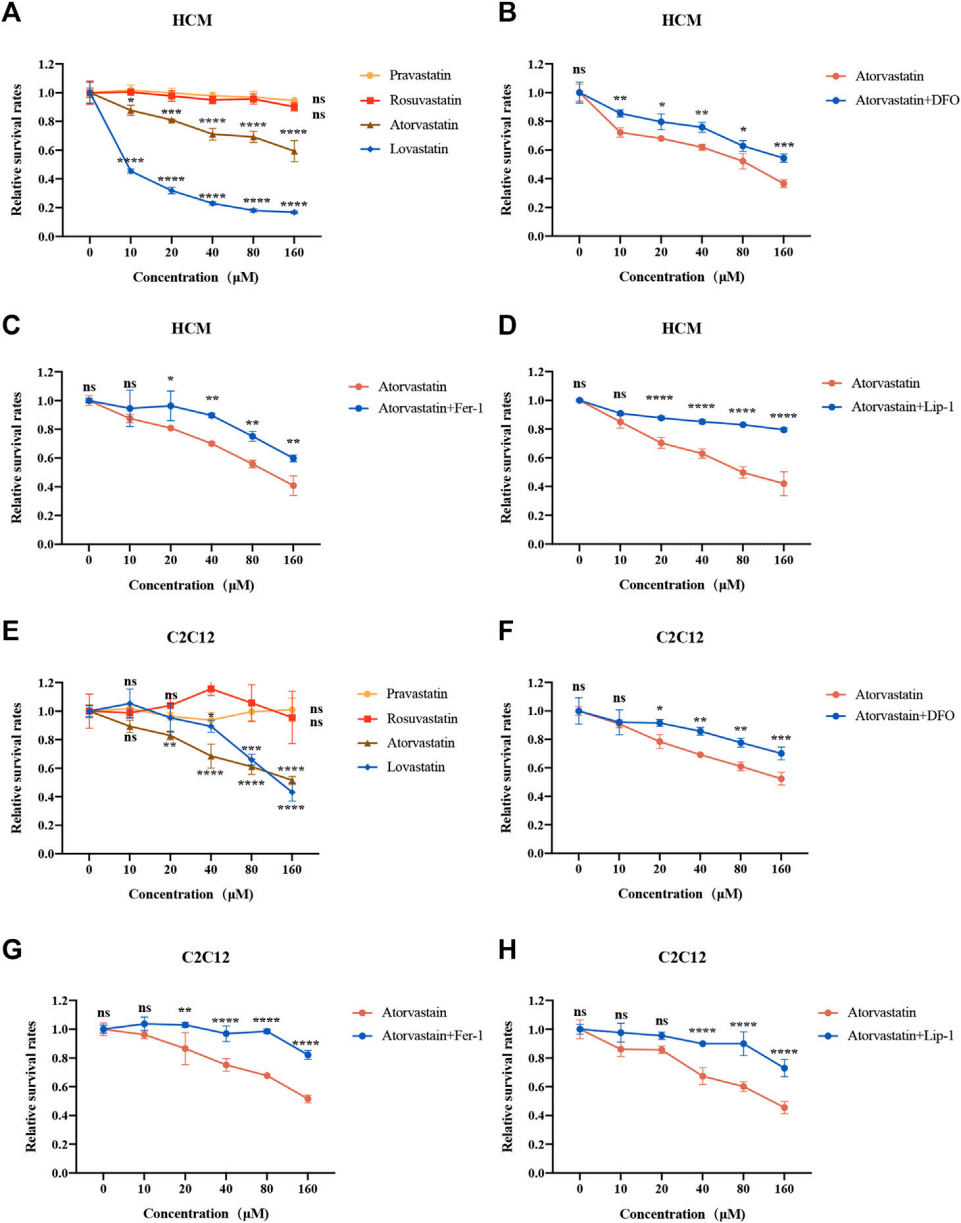
Cell viability of two kinds of muscular cell lines in response to statins for 24 h. **(A)** HCM intervened with increasing concentrations of statins were analyzed by CCK8. **(B–D)** HCM were pretreated with 100 μM DFO, 1 μM Fer-1 or 1 μM Lip-1 for 2 h, followed by the addition of increasing concentration (0–160 μM) of atorvastatin for 24 h and analyzed by CCK-8. **(E)** C2C12 cells intervened with increasing concentrations of statins for 24 h were analyzed by Cell Counting Kit-8 (CCK-8). **(F–H)** C2C12 were pretreated with 100 μM DFO, 1 μM Fer-1 or 1 μM Lip-1 for 2 h, followed by the addition of increasing concentration of atorvastatin for 24 h and analyzed by CCK-8. Data are shown as mean ± SD. ns, no significant; *p < 0.05, **p < 0.01, ***p < 0.001, ****p < 0.0001 vs. the control group. *n* = 3.

Above all, we detected the cell viability of statins in different cell lines. As shown in Figures 1A,E and Supplementary Figure S1A, atorvastatin and lovastatin reduced cell viability with increasing concentrations of statins in HCM, C2C12 and HUVEC, while rosuvastatin and pravastatin did not (Figures 1A,E and Supplementary Figure S1A). In order to explore whether restriction of ferroptosis could prevent the cell damage induced by atorvastatin and lovastatin, the cells were preprocessed with DFO, Fer-1 or Lip-1 for 2 h and cocultured with atorvastatin or lovastatin for another 24 h. Interestingly, only cell death caused by atorvastatin can be restrained by the treatment with ferroptosis inhibitor, such as DFO (Figures 1B,F), Fer-1 (Figures 1C,G) and Lip-1 (Figures 1D,H), while cell death caused by lovastatin cannot be rescued by Fer-1 in HCM and C2C12 (Supplementary Figures S2A,B). As in HUVEC cell lines, only atorvastatin and lovastatin could cause cell death (Supplementary Figure S1A), but the cell death induced by atorvastatin or lovastatin could not be rescued by ferroptosis inhibitors (Supplementary Figures S1B,C). Results suggested that ferroptosis is related to atorvastatin-induced cell injury, which was restrained within muscular cell lines. Based on the cell viability data, the concentration of 40 μM statins was selected for the deeper research. As anterior studies present that atorvastatin induces apoptosis, we also intervened HCM and C2C12 cells with caspase inhibitor Z-VAD-FMK along with atorvastatin. But our findings were inconsistent with those reported previously as the cell death damaged by atorvastatin could not be rescued by apoptosis inhibitor Z-VAD-FMK (Supplementary Figures S3A,B).

### Atorvastatin Exposure Leads to Iron Overload in HCM and C2C12 Cells

**FIGURE 2:**
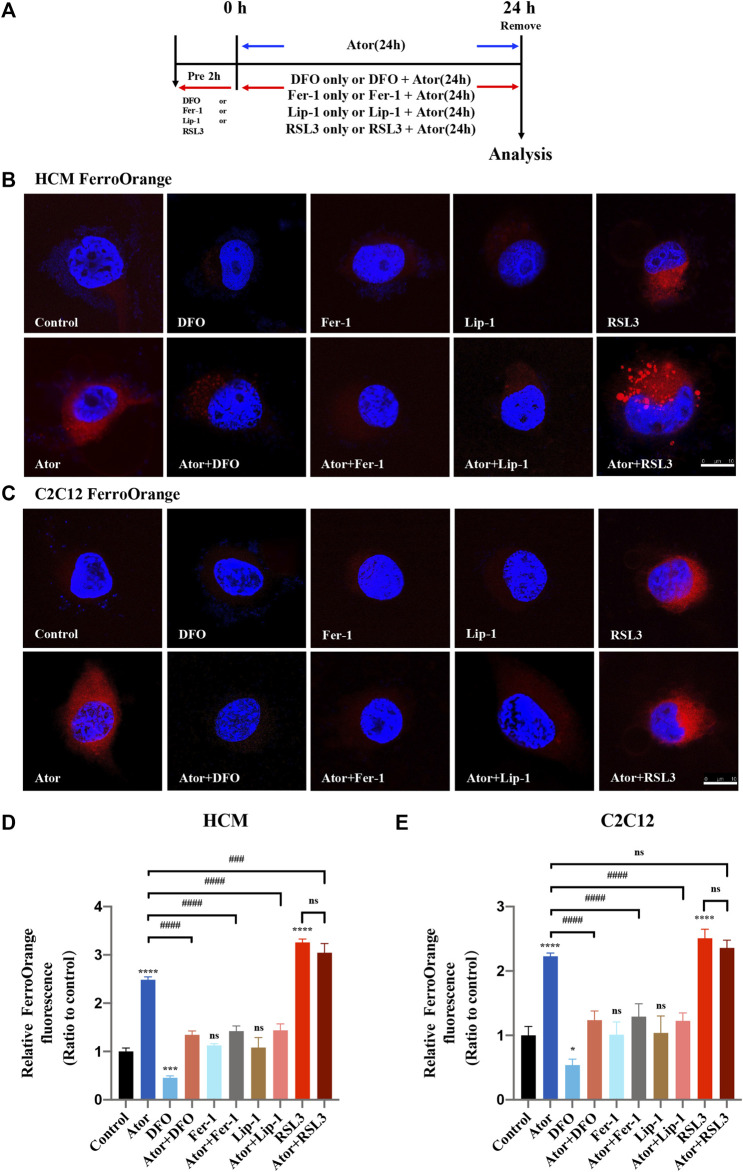
Iron accumulation in atorvastatin-supplemented HCM and C2C12 cells. **(A)** Experimental workflow. The muscular cells were pretreated with 100 μM DFO, 1 μM Fer-1, 1 μM Lip-1 or 0.5 μM RSL3 for 2 h and then intervened with or without 40 μM atorvastatin for another 24 h, or treated with 40 μM atorvastatin alone for 24 h. **(B)** Representative images of HCM treated by atorvastatin alone or with ferroptosis inhibitors DFO, Fer-1 and Lip-1 stained with FerroOrange, an indicator of intracellular Fe^2+^. **(C)** Representative images of C2C12 stained with FerroOrange. **(D)** Quantification of relative fluorescence intensity of FerroOrange in HCM. **(E)** Quantification of relative fluorescence intensity of FerroOrange in C2C12. Fluorescence intensity was quantified by ImageJ software. The data are shown as mean ± SD. ns, no significant; *p < 0.05, ***p < 0.001, ****p < 0.0001 vs. the control group; ^###^ *p* < 0.001, ^####^ *p* < 0.0001 vs*.* the atorvastatin group. *n* = 3. Scale bar: 10 μm.

Intracellular iron overload is a characteristic of cellular ferroptosis; therefore, we detected the level of intracellular iron using FerroOrange probes. The muscular cells were pretreated with RSL3, DFO, Fer-1 or Lip-1 and then intervened with or without 40 μM atorvastatin for another 24 h, or treated with 40 μM atorvastatin alone for 24 h (Figure 2A). Similar to the ferroptosis inducer RSL3 (Sui et al., 2018), atorvastatin could raise excessive iron content in HCM (approximately 2.5 times than that of the control group; *p* < 0.0001) and C2C12 cells (approximately twofold compared with that of the control group; *p* < 0.0001), as indicated by higher FerroOrange signals than those in the control groups. Interestingly, these iron accumulations can be remarkably alleviated by ferroptosis inhibitors, such as DFO (about 50%; *p* < 0.0001), Fer-1 (about 48%; *p* < 0.0001) and Lip-1 (about 48%; *p* < 0.0001) in HCM (Figures 2B,D), and the rescue effects reappeared as well in C2C12 using DFO (about 50%; *p* < 0.0001), Fer-1 (about 40%; *p* < 0.0001) and Lip-1 (about 50%; *p* < 0.0001) (Figures 2C,E). However, pretreated and cocultured with RSL3 groups compared with the atorvastatin alone group have no statistical difference in both HCM and C2C12 cells. These results indicated that overload iron was related to the atorvastatin-induced injury in HCM and C2C12 cells, and the inhibition of ferroptosis could ameliorate it.

### Atorvastatin Causes Redox Imbalance in HCM and C2C12 Cells

**FIGURE 3:**
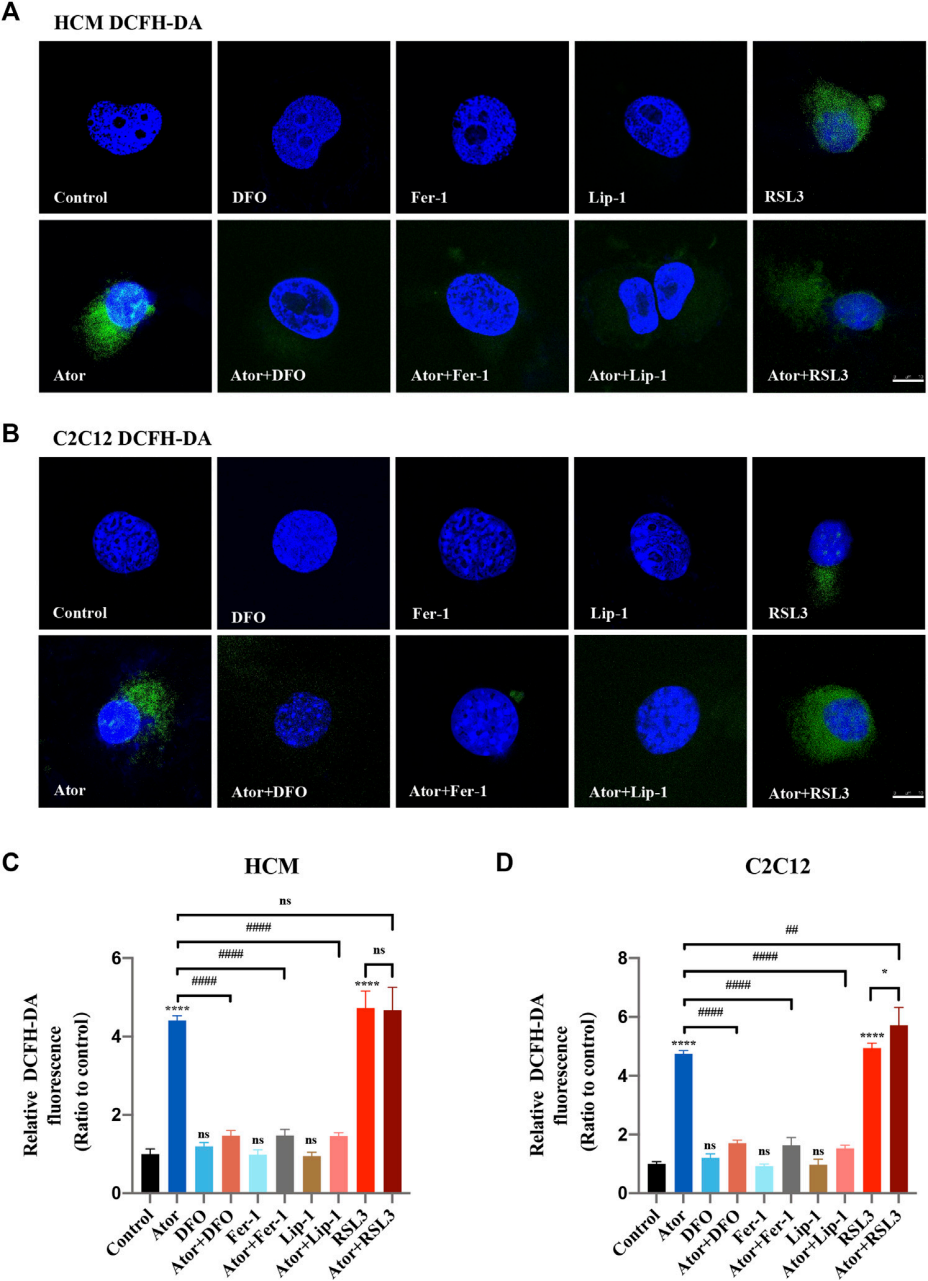
Reactive oxygen species (ROS) generation in atorvastatin-supplemented HCM and C2C12 cells. **(A)** Representative images of HCM treated by atorvastatin alone or with ferroptosis inhibitors DFO, Fer-1 and Lip-1 stained with DCFH-DA, an indicator of intracellular ROS. **(B)** Representative images of C2C12 stained with DCFH-DA. **(C)** Quantification of relative fluorescence intensity of DCFH-DA in HCM. **(D)** Quantification of relative fluorescence intensity of DCFH-DA in C2C12. The fluorescence intensity was quantified by ImageJ software. The data are shown as mean ± SD. ns, no significant; *p < 0.05, ****p < 0.0001 *vs*. the control group; ^##^ *p* < 0.01, ^####^ *p* < 0.0001 vs. the atorvastatin group. *n* = 3. Scale bar: 10 μm.

Since overloading the iron-mediated Fenton reaction followed by the ROS accumulation during ferroptosis process (Dixon et al., 2012), we then used the DCFH-DA reagent to measure the production of total ROS (Kalyanaraman et al., 2012). The staining analysis demonstrated that the ROS level was significantly increased after atorvastatin treatment in HCM (approximately fourfold compared with that of the control group; *p* < 0.0001) (Figures 3A,C) and C2C12 (approximately quintuple compared with that of the control group; *p* < 0.0001) (Figures 3B,D) cells, which restrained by ferroptosis inhibitors (about 60%; *p* < 0.0001). When atorvastatin cocultured with RSL3, they appear to act synergistically in C2C12, but it was not enough for the synergy in HCM (Figure 3).

### Ferroptosis Inhibitors Lightened Atorvastatin-Induced Lipid Peroxidation Accumulation in HCM and C2C12 Cells

**FIGURE 4:**
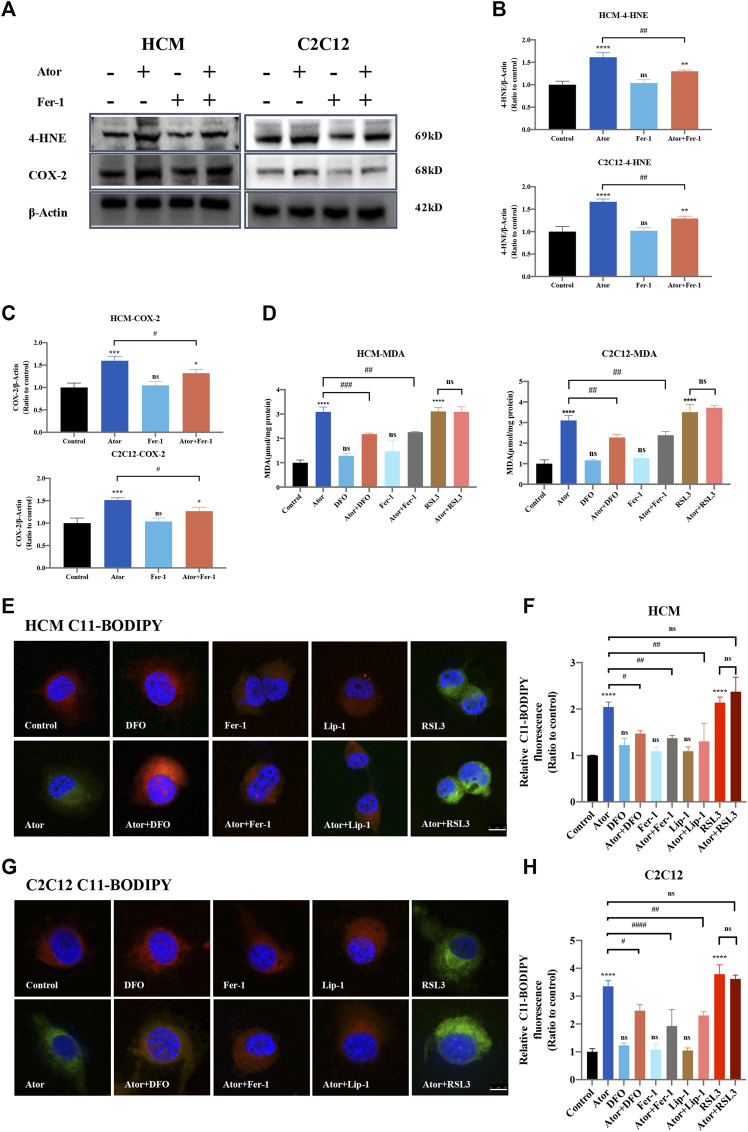
Inhibition of ferroptosis lightened atorvastatin-induced lipid peroxidation accumulation in HCM and C2C12 cells. **(A)** Western blot detection of 4-HNE and COX-2 expression in cells treated with Fer-1 and 40 μM atorvastatin or alone for 24 h. **(B,C)** Quantification of shown blot of HCM and C2C12 in **(A)**. **(D)** MDA content in HCM and C2C12 in response to atorvastatin along or with ferroptosis inhibitors DFO, Fer-1 and RSL3 for 24 h detected by the TBA test. The MDA level was expressed in μmol/mg protein. **(E)** C11BODIPY581/591 was used as the signal of lipid peroxidation to detect the effect of atorvastatin alone or with ferroptosis inhibitors DFO, Fer-1 and Lip-1 in HCM. Red images are representative of non-oxidized lipid, while the green represent oxidized lipid images of muscular cells labeled with C11-BODIPY581/591. **(F)** Relative fluorescence intensity (Green) of intracellular lipid peroxidation in HCM. Quantification of **(E)**. **(G,H)** Lipid peroxidation and the relative fluorescence intensity (green) of intracellular lipid peroxidation in C2C12. Fluorescence intensity was quantified by ImageJ software. The data are shown as mean ± SD. ns, no significant; *p < 0.05, **p < 0.01, ***p < 0.001, ****p < 0.0001 vs*.* the control group; ^#^ *p* < 0.05, ^##^ *p* < 0.01, ^###^ *p* < 0.001, ^####^ *p* < 0.0001 vs. the atorvastatin group. *n* = 3. Scale bar: 10 μm.

The expression of PTGS2/COX-2 and 4-HNE, which work as biomarkers for ferroptosis (Hu et al., 2020; Li et al., 2020), increased (approximately 1.5 times than those of the control group; *p* < 0.001) in response to atorvastatin treatment. Furthermore, ferroptosis inhibitors Fer-1 could partly lessen the atorvastatin-induced PTGS2/COX2 (about 20%; *p* < 0.05) or 4HNE expression (about 20%; *p* < 0.01) (Figures 4A–C). In addition, there was an increase in MDA, the most general byproduct of lipid peroxidation (Ghosh et al., 2019), from whole cell lysates in atorvastatin-treated HCM and C2C12, and DFO or Fer-1 significantly decreased the level that increased by atorvastatin (Figure 4D). We also observed an increase in the BODIPY581/591-C11 signal (Drummen et al., 2002) in atorvastatin-treated cells, manifesting that atorvastatin could cause lipid peroxidation in HCM (about twofold of the control group; *p* < 0.0001) and C2C12 (more than threefold of the control group; *p* < 0.0001) cells. Meanwhile, DFO, Fer-1 and Lip-1 could significantly decrease the lipid peroxidation compared to atorvastatin alone both in HCM (Figures 4E,F) and C2C12 (Figures 4G,H) represented by the BODIPY581/591-C11 signal. Moreover, treatment with DFO, Fer-1 or Lip-1 alone was unable to produce an increase or decrease in the BODIPY581/591 C11 signal and MDA content, and pretreatment and coculturing with RSL3 compared with the atorvastatin alone group have no statistical difference in both HCM and C2C12 cells. Altogether, lipid peroxidation levels increased after atorvastatin treatment, and lethal lipid peroxidation might serve as a critical medium of atorvastatin correlative muscular injury. However, the MDA content was not increased in other types of statins like lovastatin, pravastatin, and rosuvastatin, which further confirmed the conclusion that only atorvastatin leads to ferroptosis. In addition, the MDA content of other types of statins did not have a significant change when Fer-1 was added (Supplementary Figure S4).

### Atorvastatin-Induced Ferroptosis is Triggered in Mitochondria

**FIGURE 5:**
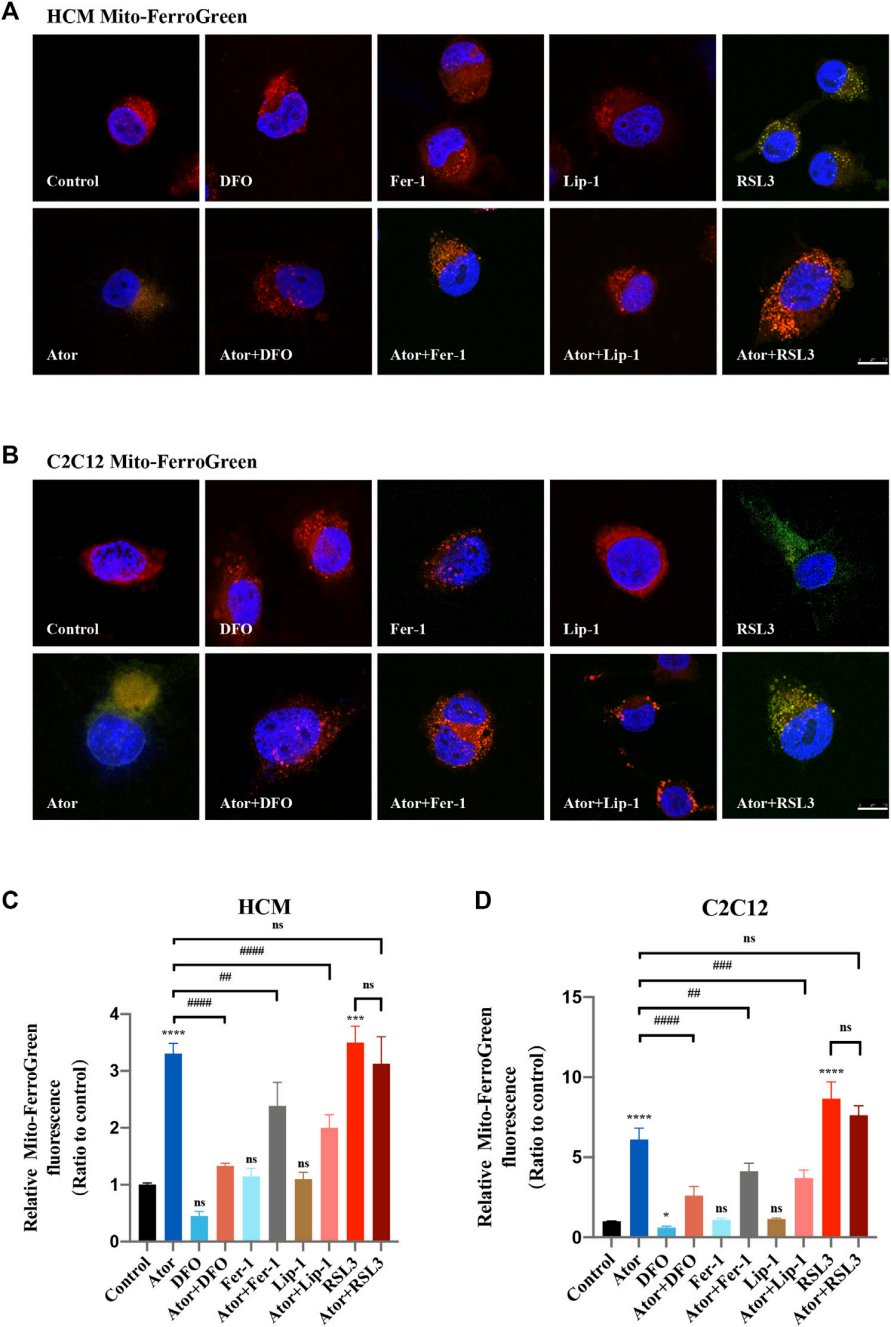
Mitochondrial iron accumulation of atorvastatin exposure in HCM and C2C12 cells. Mitochondrial Fe^2+^ analyzed by Mito-FerroGreen. Treated cell were cocultured with the probe Mito-FerroGreen for 30 min. Red images represent mitochondrial localization using MitoTracker Red CMXRos staining. **(A)** Representative images of HCM treated atorvastatin alone or with ferroptosis inhibitors DFO, Fer-1 and Lip-1 stained with Mito-FerroGreen, an indicator of mitochondrial Fe^2+^. **(B)** Representative images of C2C12 stained with Mito-FerroGreen. **(C,D)** Quantification of relative fluorescence intensity of Mito-FerroGreen in HCM and C2C12 cells. Fluorescence intensity was quantified by ImageJ software. The data are shown as mean ± SD. ns, no significant; ***p < 0.001, ****p < 0.0001 vs. the control group; ^##^ *p* < 0.01, ^####^ *p* < 0.0001 vs. the atorvastatin group. *n* = 3. Scale bar: 10 μm.

**FIGURE 6:**
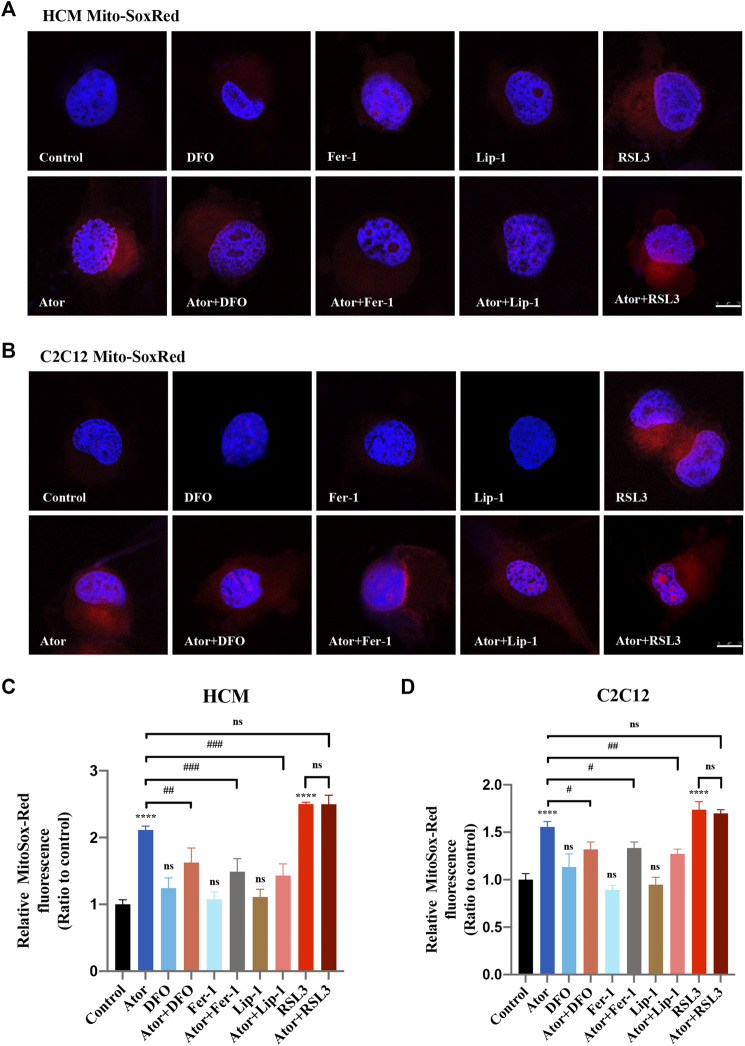
Mitochondrial ROS accumulation of atorvastatin exposure in HCM and C2C12 cells. **(A)** Representative images of HCM treated with atorvastatin alone or with ferroptosis inhibitors DFO, Fer-1 and Lip-1 stained with MitoSox-Red, an indicator of mitochondrial ROS. **(B)** Representative images of C2C12 stained with MitoSox-Red. **(C, D)** Quantification of relative fluorescence intensity of MitoSox-Red in HCM and C2C12 cells. Fluorescence intensity was quantified by ImageJ software. The data are shown as mean ± SD. ns, no significant; ****p < 0.0001 vs. the control group; ^#^ *p* < 0.05, ^##^ *p* < 0.01, ^###^ *p* < 0.001 vs. the atorvastatin group. *n* = 3. Scale bar: 10 μm.

**FIGURE 7:**
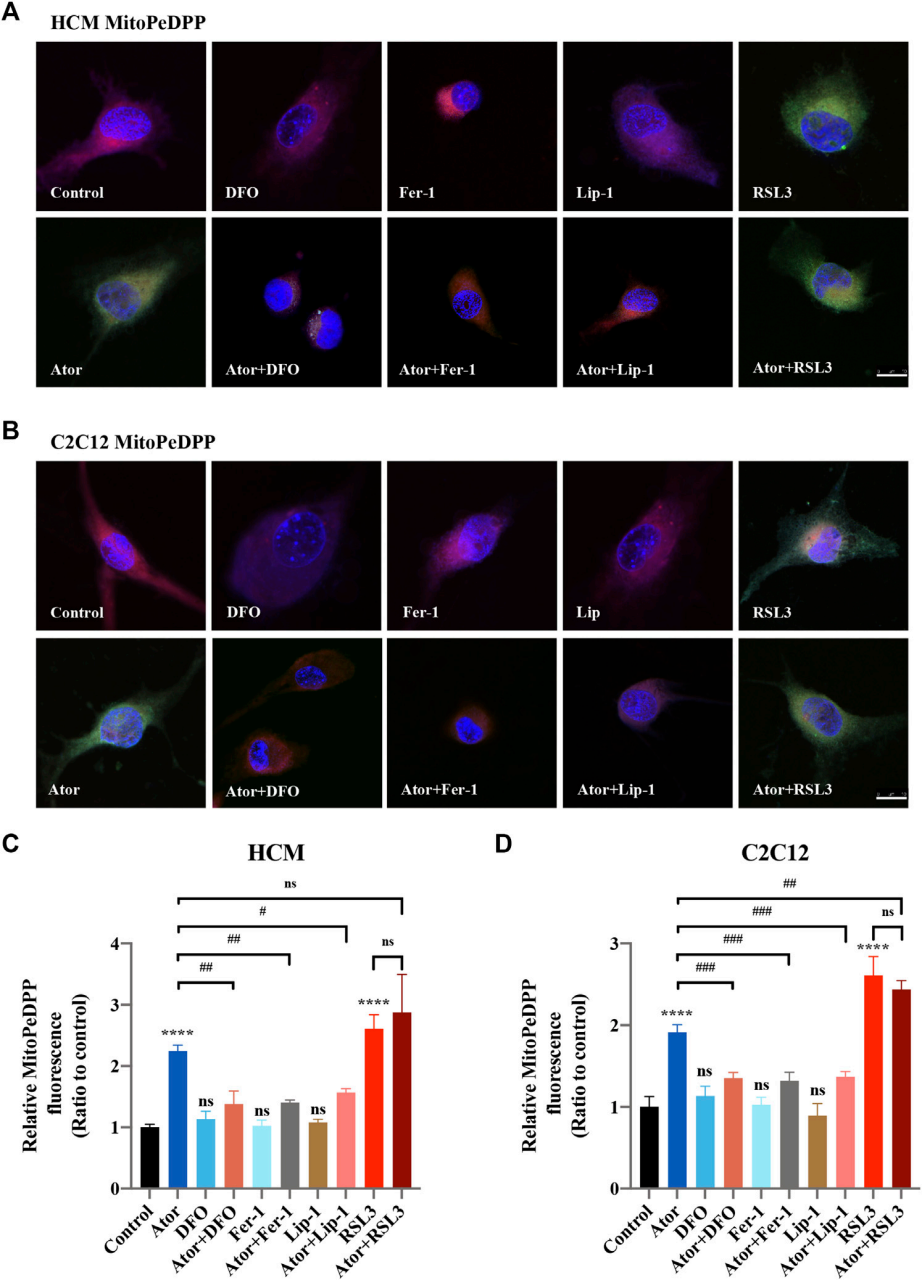
Mitochondrial lipid peroxidation of atorvastatin exposure in HCM and C2C12 cells. Using MitoDeDPP staining detected mitochondrial lipid peroxidation. Red images represent mitochondrial localization using MitoTracker Red CMXRos staining, while the green is representative of mitochondrial lipid peroxidation labeled with MitoDeDPP. **(A)** Representative images of HCM treated atorvastatin alone or with ferroptosis inhibitors DFO, Fer-1 and Lip-1 stained with MitoDeDPP. **(B)** Representative images of C2C12 stained with MitoDeDPP. **(C,D)** Quantification of relative fluorescence intensity of MitoDeDPP in HCM and C2C12 cells. Fluorescence intensity was quantified by ImageJ software. The data are shown as mean ± SD. ns, no significant; ****p < 0.0001 vs*.* the control group; ^#^ *p* < 0.05, ^##^ *p* < 0.01, ^###^ *p* < 0.001 vs. the atorvastatin group. *n* = 3. Scale bar: 10 μm.

Mitochondrial dysfunction is a crucial factor in myopathies. To analyze the role of mitochondria in atorvastatin-induced ferroptosis, mitochondrial content of Fe^2+^, ROS and lipid peroxidation were detected. Confocal microscopy results showed that the signal of Mito-FerroGreen (MFG), a mitochondria-specific Fe^2+^ fluorescence indicator, increased threefold compared with the group of atorvastatin alone in HCM (Figures 5A,C) (*p* < 0.0001), and it even reached more than fivefold in C2C12 (Figures 5B,D) (*p* < 0.0001). DFO, Fer-1 and Lip-1 lessen the trend in HCM and C2C12, respectively. Aforementioned results indicated that atorvastatin will cause free iron accumulation in mitochondria, which could be rescued by ferroptosis inhibitors. When we detected ROS in mitochondria, the condition was similar to the determination of free ferrous iron. Atorvastatin could induce ROS assembling massively in mitochondria, which was partially reduced by ferroptosis inhibitors of DFO, Fer-1 and Lip-1 in HCM (Figures 6A,C) and C2C12 (Figures 6B,D). To further investigate the role of mitochondria, we used MitoPeDPP to examine the mitochondrial lipid peroxidation. The level of mitochondrial lipid peroxidation labeled with MitoPeDPP was also significantly increased by atorvastatin (more than twofold compared with the control group; *p* < 0.0001), and the increase was rescued by ferroptosis inhibitors in HCM (Figures 7A,C). This alteration of mitochondrial lipid peroxidation was reproduced in C2C12 (Figures 7B,D). However, pretreatment and coculturing with RSL3 compared with the atorvastatin alone group had no statistical difference in both HCM and C2C12 cells, which may be because of the threshold of the reaction toward oxidative stress. The ferroptosis indicator changed significantly on mitochondria, which was not so different with what occurred intracellularly, suggesting that mitochondria may play a critical role in atorvastatin-induced ferroptosis.

### Atorvastatin-Induced Morphological Change of Mitochondria and Breakdown of MMP in HCM and C2C12 Cells

**FIGURE 8:**
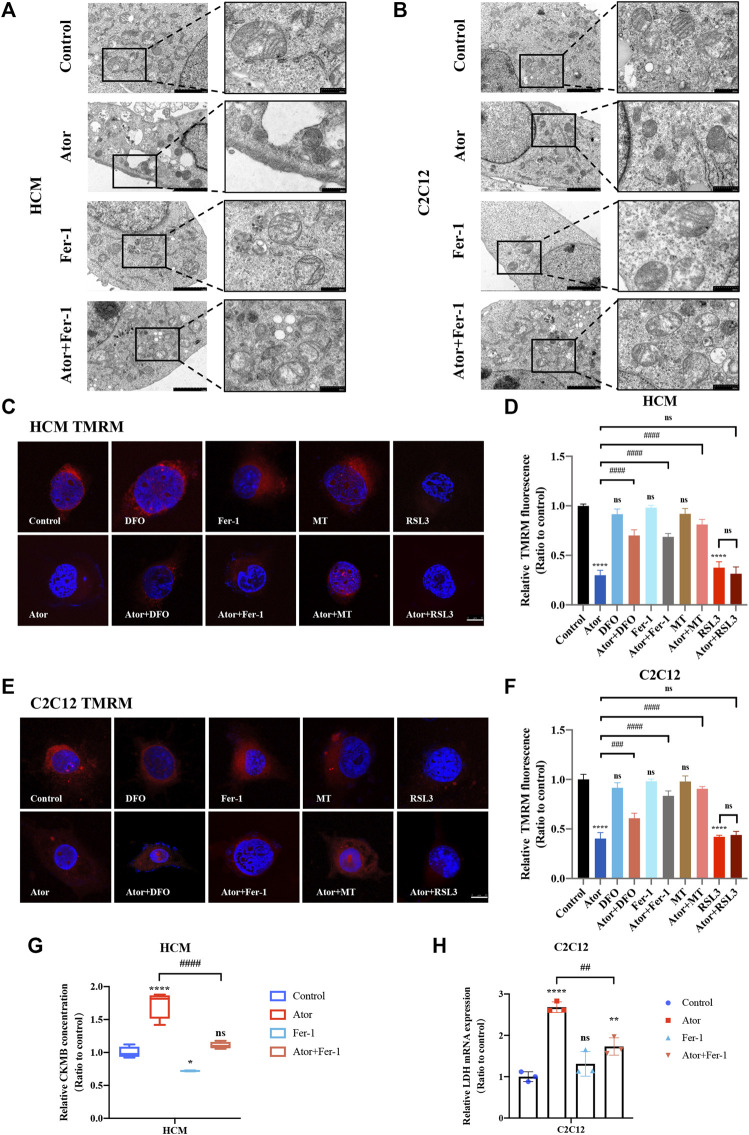
Morphological changes of mitochondria and breakdown of MMP in atorvastatin treated HCM and C2C12 cells. **(A,B)** Transmission electron microscopy images of mitochondria ultrastructure in both HCM and C2C12 cells. Scale bar in left lower inset: 2 μM, right lower inset: 500 nm. **(C)** Representative images of HCM treated atorvastatin alone or with ferroptosis inhibitors DFO, Fer-1 and MT stained with TMRM, an indicator of mitochondrial membrane potential. **(D)** Relative fluorescence intensity of TMRM in HCM. Quantification of **(C)**. **(E,F)** Representative images and relative fluorescence intensity of TMRM in C2C12. **(G)** Relative levels of CKMB in HCM treated by atorvastatin alone or with Fer-1 examined by ELISA. **(H)** Relative mRNA expression of LDH in C2C12 treated by atorvastatin alone or with Fer-1. The fluorescence intensity was quantified by ImageJ software. The data are shown as mean ± SD. ns, no significant; *p < 0.05, **p < 0.01, ****p < 0.0001 vs. the control group; ^#^ *p* < 0.05, ^##^ *p* < 0.01, ^###^ *p* < 0.001, ^####^ *p* < 0.0001 vs. the atorvastatin group. *n* = 3. Scale bar: 10 μm.

To assess the morphologic features, we used a transmission electron microscope to observe the change of the ultra-microstructure. Shrunk mitochondria with increased membrane density were observed in atorvastatin-treated cells (Figures 8A,B). Fer-1 treatment remarkably ameliorated atorvastatin-induced mitochondrial abnormality, which is consistent with ROS and lipid peroxidation changes in mitochondria of muscular cells. These data provide convincing evidence of the occurrence of atorvastatin-induced ferroptosis in mitochondria of muscular cells. To further verify the effects on mitochondria, we checked the effects of MMP (represents mitochondrial function) by TMRM staining in atorvastatin-supplemented HCM and C2C12 cells. From Figure 8, TMRM fluorescence decreased significantly after atorvastatin treatment, manifesting that atorvastatin caused a breakdown of membrane potential. The inhibition of ferroptosis by Fer-1 and DFO could recover the abnormal mitochondrial membrane potential caused by atorvastatin. Moreover, mitochondria-targeted antioxidant MitoTEMPO (MT) treatment not only shielded the cells from mitochondrial damage after exposure of atorvastatin as a recovery of TMRM fluorescence intensity appeared in HCM (Figures 8C,D) and in C2C12 (Figures 8E,F) but also significantly prevented atorvastatin-induced myocyte death (Supplementary Figures S5A,B). Also, pretreatment and coculturing with RSL3 compared with the atorvastatin alone group have no statistical difference toward TMRM in both HCM and C2C12 cells. In general, atorvastatin-induced mitochondria-dependent ferroptosis accompanied with mitochondrial dysfunction is one of the basic matters within muscular cell damage.

### Inhibition of Ferroptosis-Lightened Atorvastatin-Induced Myocardial Damage and Muscular Injury in HCM and C2C12 Cells

Creatine kinase-MB (CKMB) works as a representative of myocardial injury biomarker in several cardiovascular conditions like sepsis and acute myocardial infarction (AMI) (Saenger and Jaffe, 2008; Ndrepepa and Kastrati, 2018). To evaluate the myocyte injury potential of atorvastatin, cell free supernatant levels of CKMB were detected by enzyme-linked immunosorbent assay (ELISA). The results indicated that CKMB increased in the atorvastatin group (*p* < 0.0001), which could be restrained by fer-1 to some extents in HCM (*p* < 0.0001). As for skeleton muscle, lactate dehydrogenase (LDH) was selected on behalf of the musculoskeletal injury marker (Milias et al., 2005; Bazzucchi et al., 2019). Elevated LDH after atorvastatin exposure in C2C12 cells (more than two times of the control group; *p* < 0.0001) was also partially ameliorated by fer-1(about 38%; *p* < 0.01) (Figures 8G,H). In summary, the inhibition of ferroptosis-alleviated atorvastatin-induced myocardial damage and muscular injury in HCM and C2C12 cells, and ferroptosis was at least partly involved in atorvastatin-caused cell injury.

### Nrf2-xCT/GPX4 Pathway Involved in Ferroptosis Induced by Atorvastatin

**FIGURE 9:**
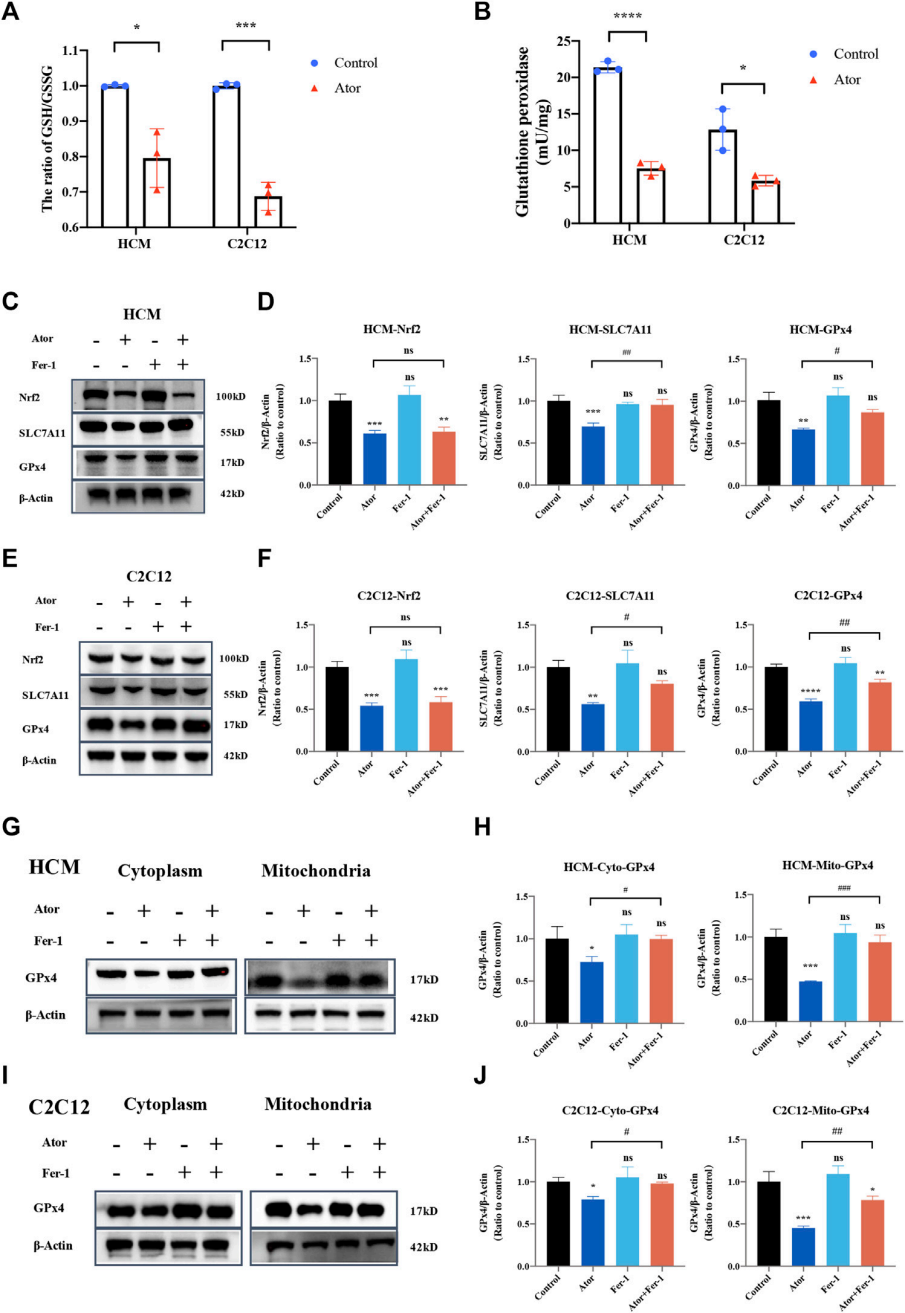
Dysregulation of Nrf2-GPx4/xCT pathway was related to ferroptosis induced by atorvastatin. **(A)** Ratio of GSH/GSSG in lysates from the cultured myocytes. **(B)** Glutathione peroxidase activity in lysates from the cultured myocytes. **(C, D)** Expression of proteins of Nrf2, SLC7A11, and GPx4 in cultured HCM treated by atorvastatin alone or with Fer-1 detected by Western blot. **(E,F)** Expression of proteins of Nrf2, SLC7A11, and GPx4 in cultured C2C12 detected by Western blot. **(G,H)** Expression of GPx4 protein in cultured HCM atorvastatin-treated alone or with Fer-1 in cytoplasm (without mitochondria) or mitochondria detected by Western blot. **(I,J)** Expression of GPx4 protein in cultured C2C12 atorvastatin-treated alone or with Fer-1 in cytoplasm (without mitochondria) or mitochondria in C2C12 detected by Western blot. The fluorescence intensity was quantified by ImageJ software. The data are shown as mean ± SD. ns, no significant; *p < 0.05, **p < 0.01, ***p < 0.001, ****p < 0.0001 vs. the control group; ^#^ *p* < 0.05, ^##^ *p* < 0.01, ^###^ *p* < 0.001 vs. the atorvastatin group. *n* = 3. Scale bar: 10 μm.

In order to investigate the mechanism underlying atorvastatin-induced ferroptosis, the GSH/GSSG ratio was tested. As expected, the GSH/GSSG ratio was decreased significantly both in HCM and C2C12 (Figure 9A). In addition, glutathione peroxidase (Figure 9B) and the expression of GPx4 were downregulated by atorvastatin treatment compared with the control group in HCM (Figures 9C,D) and C2C12 (Figures 9E,F). As well as the expression of SLC7A11 and Nrf2 was decreased by atorvastatin. Fer-1 could elevate the expression of SLC7A11 and GPx4 compared with that of the atorvastatin alone group, but it did not influence the levels of Nrf2 (Figures 9C–F). Aforementioned results imply that the Nrf2-xCT/GPx4 signaling pathway might be involved in atorvastatin-induced ferroptosis. When we isolated mitochondria from cytoplasm, GPx4 was decreased much more significantly in mitochondria (about 60%; *p* < 0.001) than other parts of organelles that exist in cytoplasm (about 20–30%; *p* < 0.05). In mitochondria, the expression of GPx4 was slightly but significantly rescued by ferroptosis inhibitor Fer-1 as well when compared with the single atorvastatin-treated group (Figures 9G–J). The results suggested that mitochondria GPx4 may be a key point in pathogenesis of ferroptosis caused by atorvastatin.

To investigate whether the CoQ-dependent system is also involved in atorvastatin-induced ferroptosis, we investigated the CoQ10 levels within atorvastatin exposure in HCM. Our results did not support this hypothesis since there was no statistical difference among groups of CoQ10 and dihydroorotate dehydrogenase (DHODH) (Supplementary Figures S5A–C).

## Discussion

Despite extensive application of statins, the pathogenesis of statin-induced myopathy remains largely unknown. We observed that atorvastatin administration caused oxidative stress and cell damage, which is the same as the results of hepatic tissue damage (Pal et al., 2015). In the present study, atorvastatin exposure decreased the muscular cell viability in a dose-dependent manner, which could be rescued by ferroptosis inhibitors significantly. At the same time, indicators of ferroptosis such as iron overload, ROS accumulation, and lipid peroxidation occurred in atorvastatin-treatment muscular cells, especially in mitochondria. Although some speculations conjectured that statins will provoke ferroptosis (Chen and Galluzzi, 2018; Chen et al., 2021), but there is only one study that reported that simvastatin caused cancer cell ferroptosis by suppressing the expression of HMGCR (Yao et al., 2021). We first identified that ferroptosis participates in atorvastatin-induced myopathy, providing a potential strategy to prevent atorvastatin-induced heart damage and muscle symptoms by inhibiting ferroptosis.

As it is known that the muscular injury of statins is dose dependent. The dosage selected for research is important, but the dosage of the drug taken by the human body is difficult to convert into the concentration *in vitro*, which involves the rate of absorption and metabolism (Needham and Mastaglia, 2014). Moreover, it is worth mentioning that drug interaction and potent inhibitors of cytochrome P450 (CYP) 3A4 inhibitors (atorvastatin is metabolized by CYP3A4) can significantly increase statin concentrations (Hirota et al., 2020), and clinical research on atorvastatin tends to apply high dose (Pedersen et al., 2005) since high-dose atorvastatin is superior in preventing peripheral arterial disease (Stoekenbroek et al., 2015). Therefore, the relatively high concentrations of statins are also necessary. Among the cell studies, the concentration range of statins is from nanomolar concentrations to micromolar concentrations (Obata and Nakashima, 2016; Cai and Gao, 2021). The concentrations of statins applied *in vitro* were 10 μM (Oh et al., 2020), 20 μM (Qian et al., 2019), 30 μM (Cai and Gao, 2021), 50 μM [atorvastatin was applied to study statin-induced muscle damage in C2C12 and primary murine myoblasts (Hoppstädter et al., 2020)], 60 μM [simvastatin was used to measure the effect of statins on C2C12 myotubes (Moschetti et al., 2021)], 100 μM [atorvastatin was used to investigate the protective effects against doxorubicin-induced cardiotoxicity in H9C2 (Obata and Nakashima, 2016; Li et al., 2019)], and 333.3 μM [which was used to test the efficacy and toxicity of a new hyaluronan-derived nanoparticle delivery system for simvastatin in tissue-engineered skeletal muscle (Jones et al., 2019)]. The concentration in our study is within the range, and the selected concentration of 40 μM is beneath the IC50 in both HCM and C2C12.

Moreover, we found that only atorvastatin induces ferroptosis, while lovastatin, pravastatin, or rosuvastatin did not. Actually, statins did not always act equally, and the bioequivalence of statins should not be confirmed. The common clinical goal of statins is to reduce LDL-C, but statins have various effects on many other biochemical pathways, and those effects may be commonsensical expected to differ from statins to statins (Trapani et al., 2011; Bergt et al., 2017). For example, for patients who have hypercholesterolemia, only pravastatin but not atorvastatin, could reduce the atherogenic lipoprotein-associated phospholipase A2 (Ky et al., 2008), and lovastatin, but not pravastatin, causes higher mortality of cardiomyopathic hamsters (März et al., 2000). A better comprehension of various molecular pathways is essential and will have suggestions in the decision-making process among specific patient populations.

Overwhelming accumulation of Fe^2+^, ROS and lipid peroxidation occurs in atorvastatin treatment cells in our study. As is known, ROS plays a major role in atorvastatin induced oxidative stress (Qi et al., 2013; Bouitbir et al., 2012). It contributes to the mitochondrial damage, oxidative injury of the cellular membranes, DNA damage, and so forth (Eruslanov and Kusmartsev, 2010; Bras et al., 2005; Pelicano et al., 2004). In this study, we further identified that ROS was triggered by atorvastatin-induced free iron-overload through a Fenton reaction in muscular cells. The accumulation of ROS subsequently assaulted the bio-membrane system, leading to a lipid peroxidation finally.

Furthermore, mitochondrial dysfunction, in particular impaired function of the electron transport chain, is frequently associated with in statin-associated skeletal muscle damage (Larsen et al., 2013; Bouitbir et al., 2016). We not only confirmed that atorvastatin impairs the mitochondrial function but further found that ferroptosis is involved in the mitochondrial dysfunction since it is effective to protect the mitochondrial function by ferroptosis inhibitor. And our mitochondria ultrastructure results provided convincing evidence that ferroptosis participates in the process of mitochondrial dysfunction induced by atorvastatin.

**FIGURE 10:**
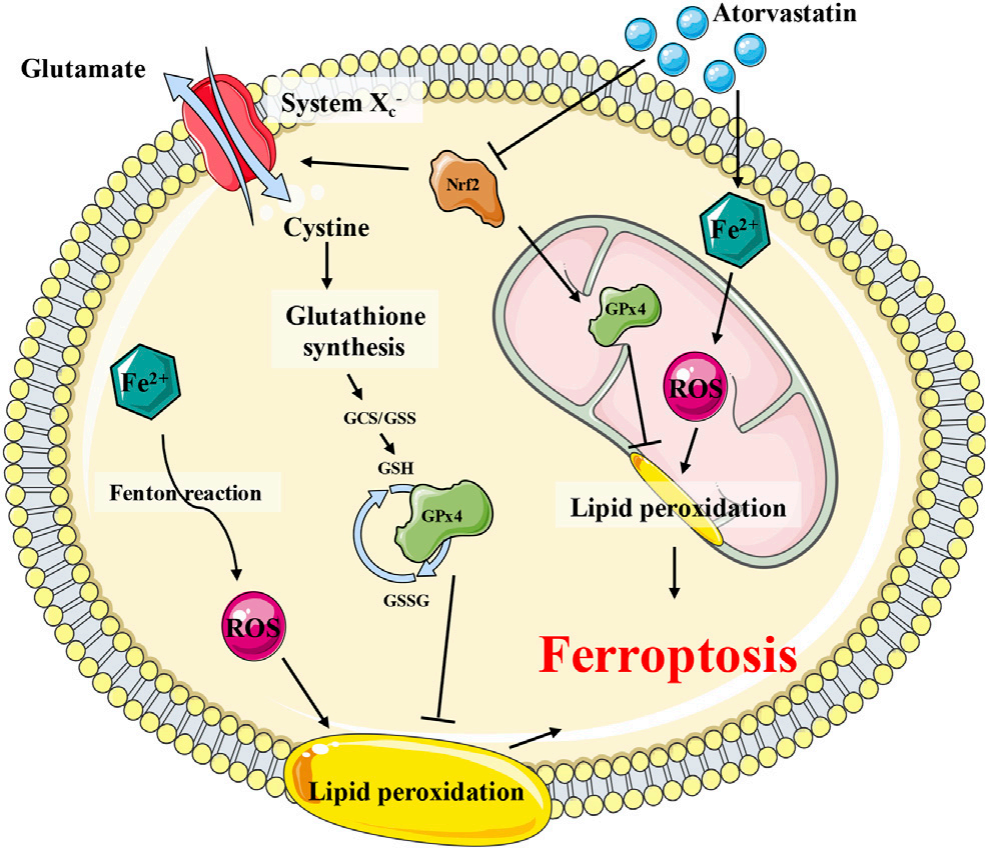
Proposed scheme for the underlying mechanisms of atorvastatin-induced muscular cell ferroptosis. Atorvastatin suppressed the Nrf2, which would, in turn, inhibit the expression of System xc− (SLC7A11) and GPx4 (especially the mitochondrial GPx4), leading to a severe damage to the antioxidant system of ferroptosis. Consistently restraining of System xc− (SLC7A11) brought on GSH depletion, which would exaggerate to the dysfunction of GPx4 as well. The GPx4 enzyme catalyzes the transformation of GSH to GSSG and at the meantime decreases the toxic peroxide to non-toxic hydroxy compounds to protect the structure and function of the cell membrane against peroxides.

GPx4 is the most critical ferroptosis defense gene that encodes cytosolic, mitochondrial, and nucleolar isoforms (Yang et al., 2014; Ingold et al., 2018), whose functions include scavenging free radicals and detoxifying various xenobiotics and, consequently, converting itself to its oxidized form, glutathione disulfide (GSSG). In the present study, following atorvastatin intoxication, the expression and function of GPx4 in the mitochondria and intracellular organelles were greatly impaired, indicating that GPx4 may play a critical role in the atorvastatin-induced ferroptosis. This result is consistent with previous study where GPx4 was downregulated as well in atorvastatin-induced liver injury (Kromer and Moosmann, 2009). Another key regulator of the GSH-dependent antioxidation system was System xc−(xCT), an amino acid antiporter that acts as an important reservoir for the supplement of GSH (Jiang et al., 2015; Wang et al., 2017; Ingold et al., 2018; Daher et al., 2019; Koppula et al., 2021). The key component of xCT, SLC7A11, was downregulated by atorvastatin. Nrf2 is the key transcription factor to maintain oxidative homeostasis and is activated under conditions of high oxidative stress, promoting target gene transcription such as GPx4, HO-1, and SLC7A11 (Shin et al., 2018; Dodson et al., 2019). In our results, Nrf2 also was downregulated by atorvastatin, implying that Nrf2 is inhibited by atorvastatin and then suppressed the GPx4 as well as SLC7A11 expression in the process of ferroptosis. Proposed scheme for the underlying mechanisms of atorvastatin-induced muscular cell ferroptosis was diaplayed in Figure 10.

Another ferroptosis-defense system is the CoQ-dependent pathway involving FSP1 and mitochondrial DHODH. In our study, CoQ10 levels of atorvastatin exposure in HCM did not have a statistical difference among groups. Therefore, the ubiquinone content may not be the explanation for the observed ferroptosis in atorvastatin treatment. In support of this assumption, it appears that in most patients treated with statins, the skeletal muscle CoQ10 content remains high enough to maintain the ETC function (Banach et al., 2015). In addition, DHODH, the key component of CoQ-dependent system which operates in parallel to mitochondrial GPx4 to inhibit ferroptosis, did not have a statistical difference among groups, either. Therefore, the CoQ-dependent metabolic pathway was not the mechanism to explain atorvastatin-induced ferroptosis, and more experiment should be conducted to confirm this deduction in the future.

Taken together, our data point toward ferroptosis as an essential molecular mechanism leading to statin-induced muscle damage, providing valuable new insights into homeostatic mechanisms required for the tolerance of the widely prescribed atorvastatin.

## Data Availability

The original contributions presented in the study are included in the article/Supplementary Material, further inquiries can be directed to the corresponding authors.
