# Supplementary figures and images for: Atorvastatin Induces Mitochondria-Dependent Ferroptosis via the Modulation of Nrf2-xCT/GPx4 Axis
Source: Front Cell Dev Biol. 2022 Mar 3;10:806081. doi: 10.3389/fcell.2022.806081 (PMC8927716; doi:10.3389/fcell.2022.806081)

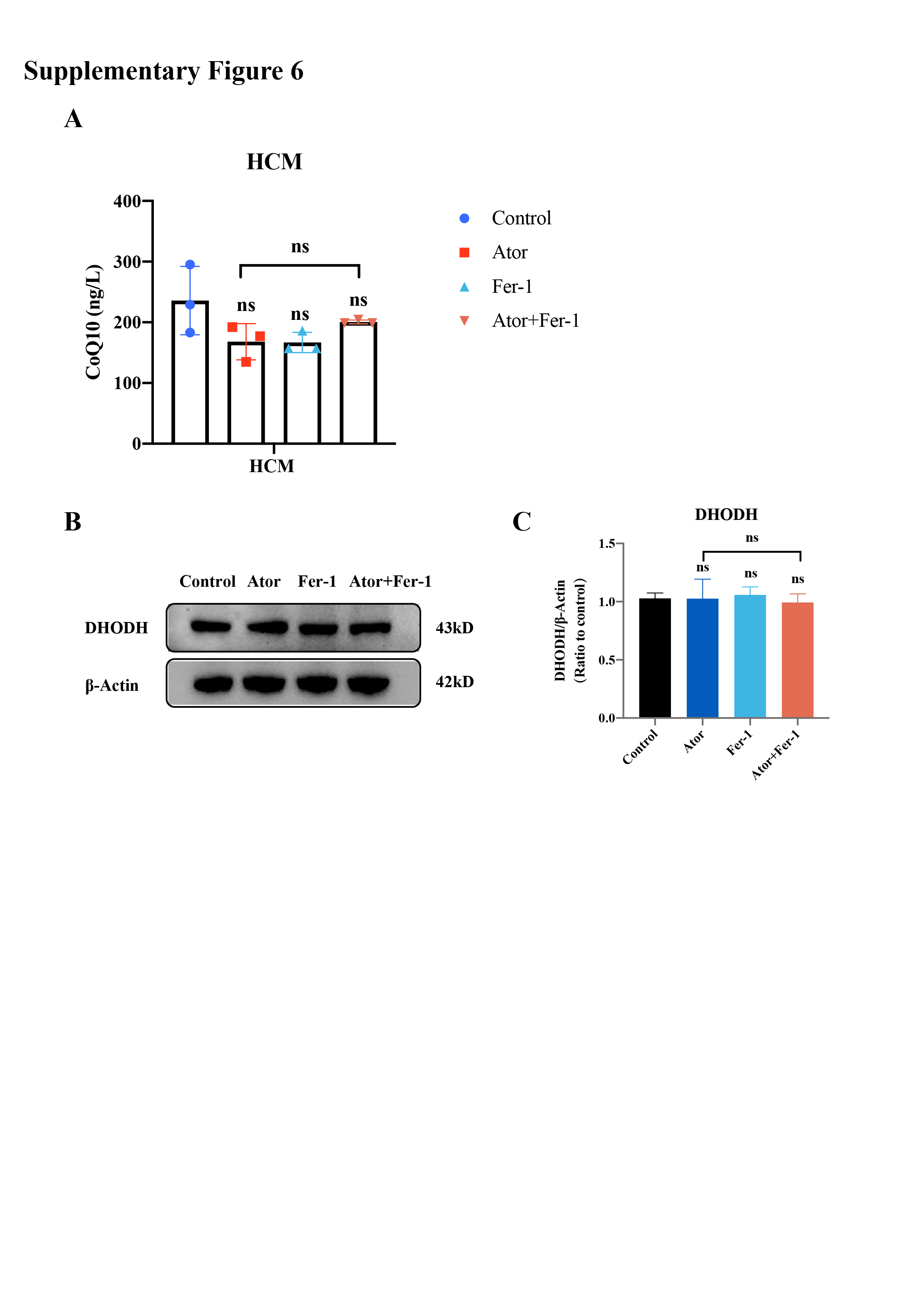

Supplement: Supplementary file 1 [file Image6.tif]

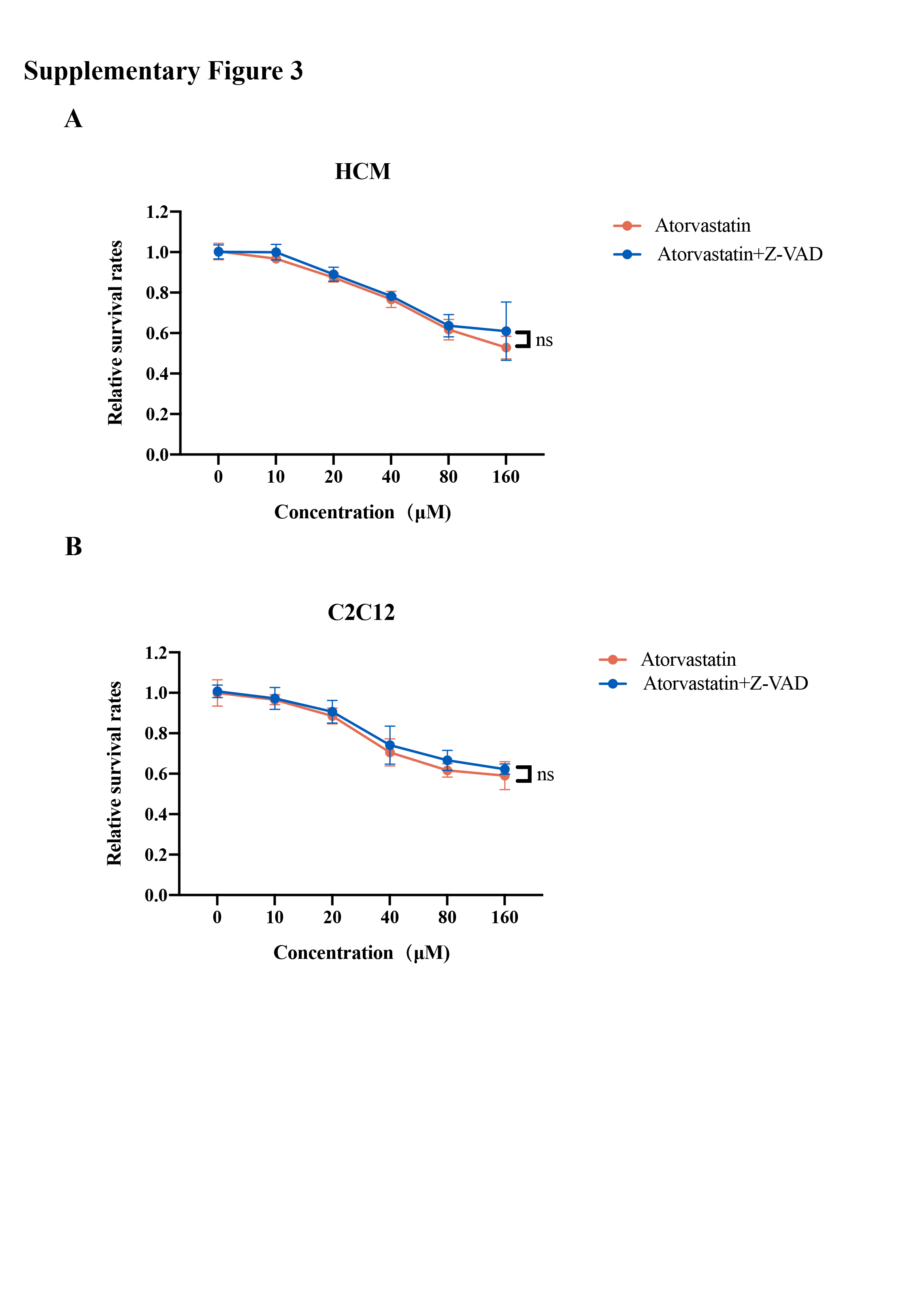

Supplement: Supplementary file 2 [file Image3.tif]

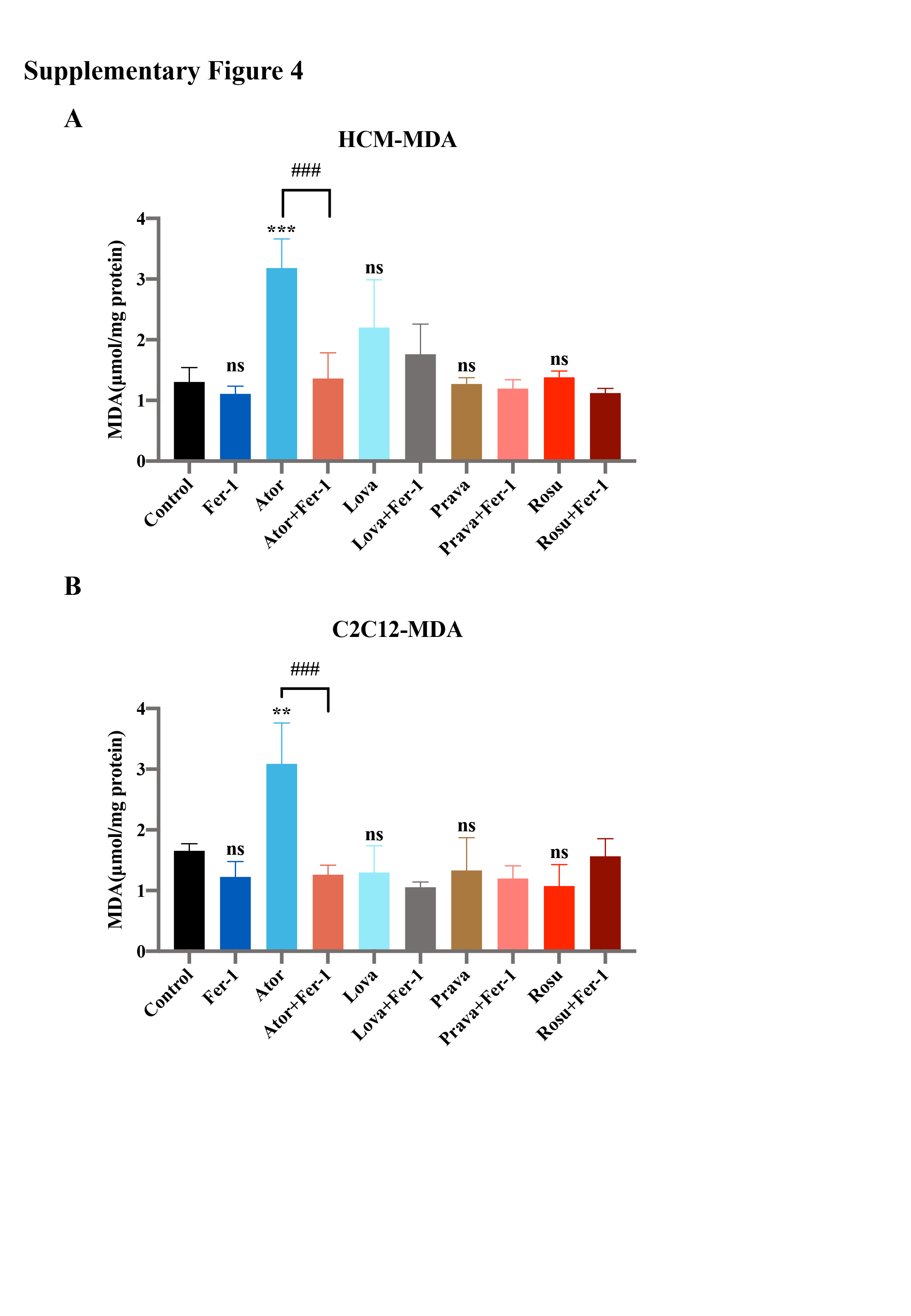

Supplement: Supplementary file 3 [file Image4.tif]

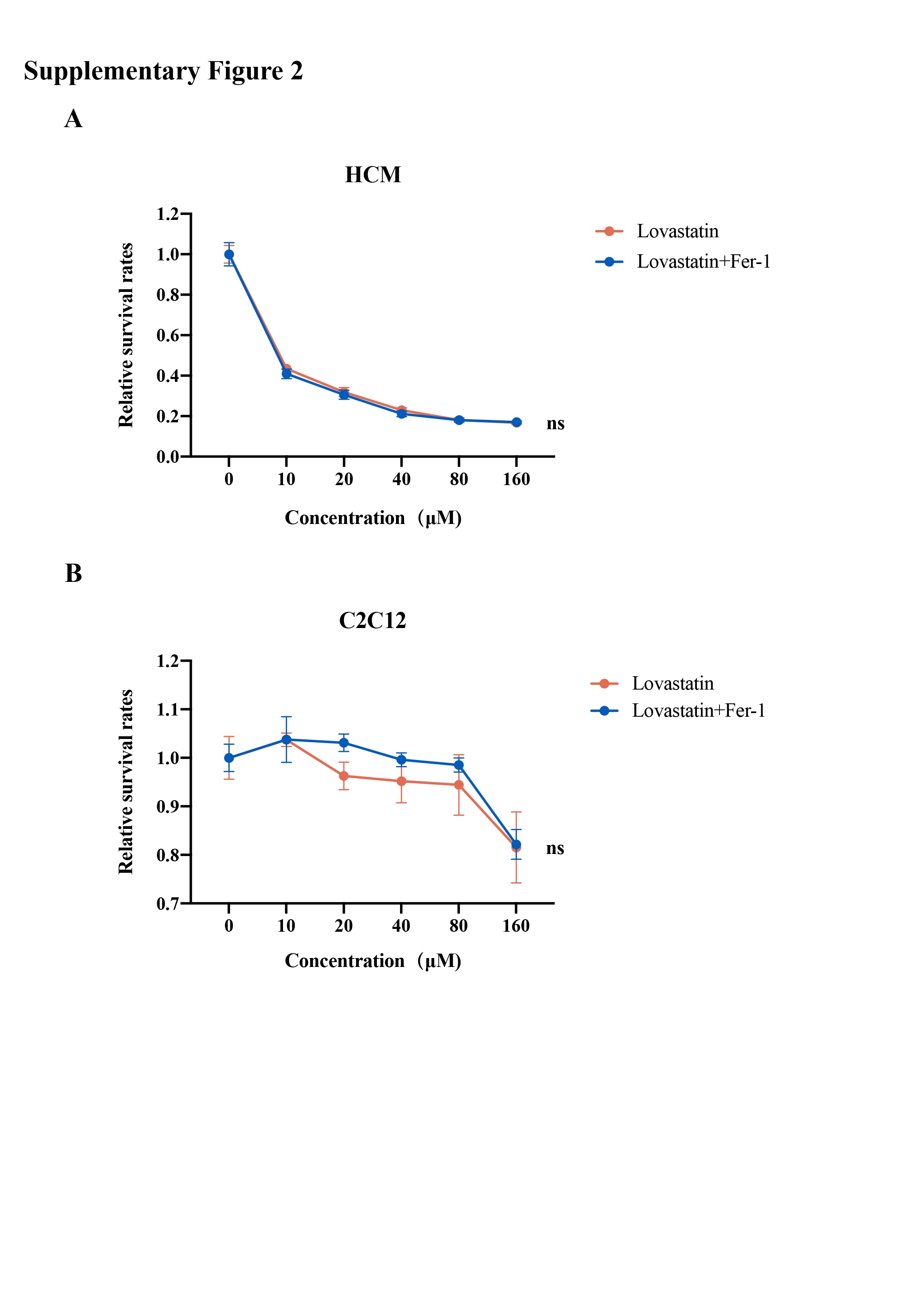

Supplement: Supplementary file 4 [file Image2.tif]

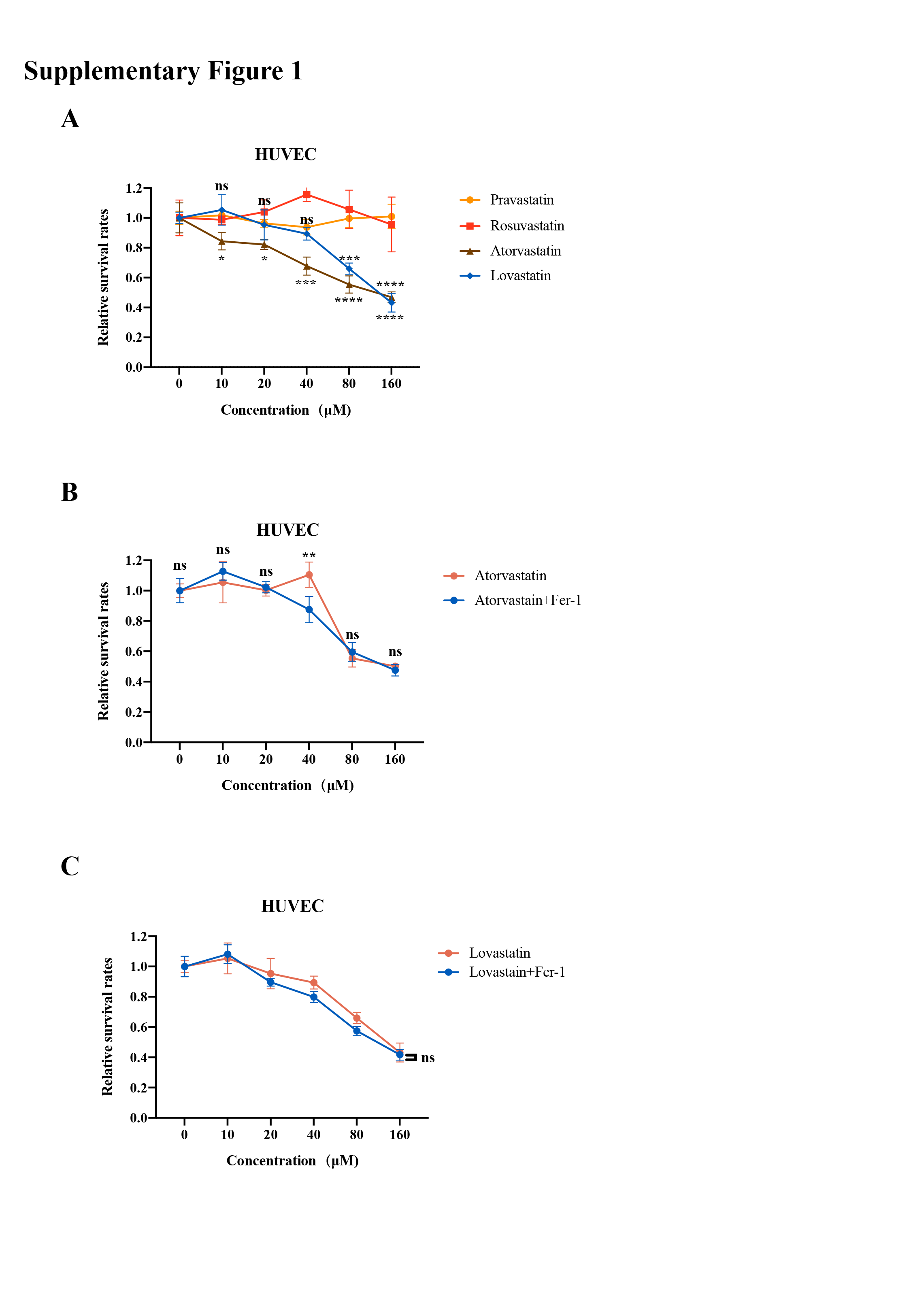

Supplement: Supplementary file 5 [file Image1.tif]

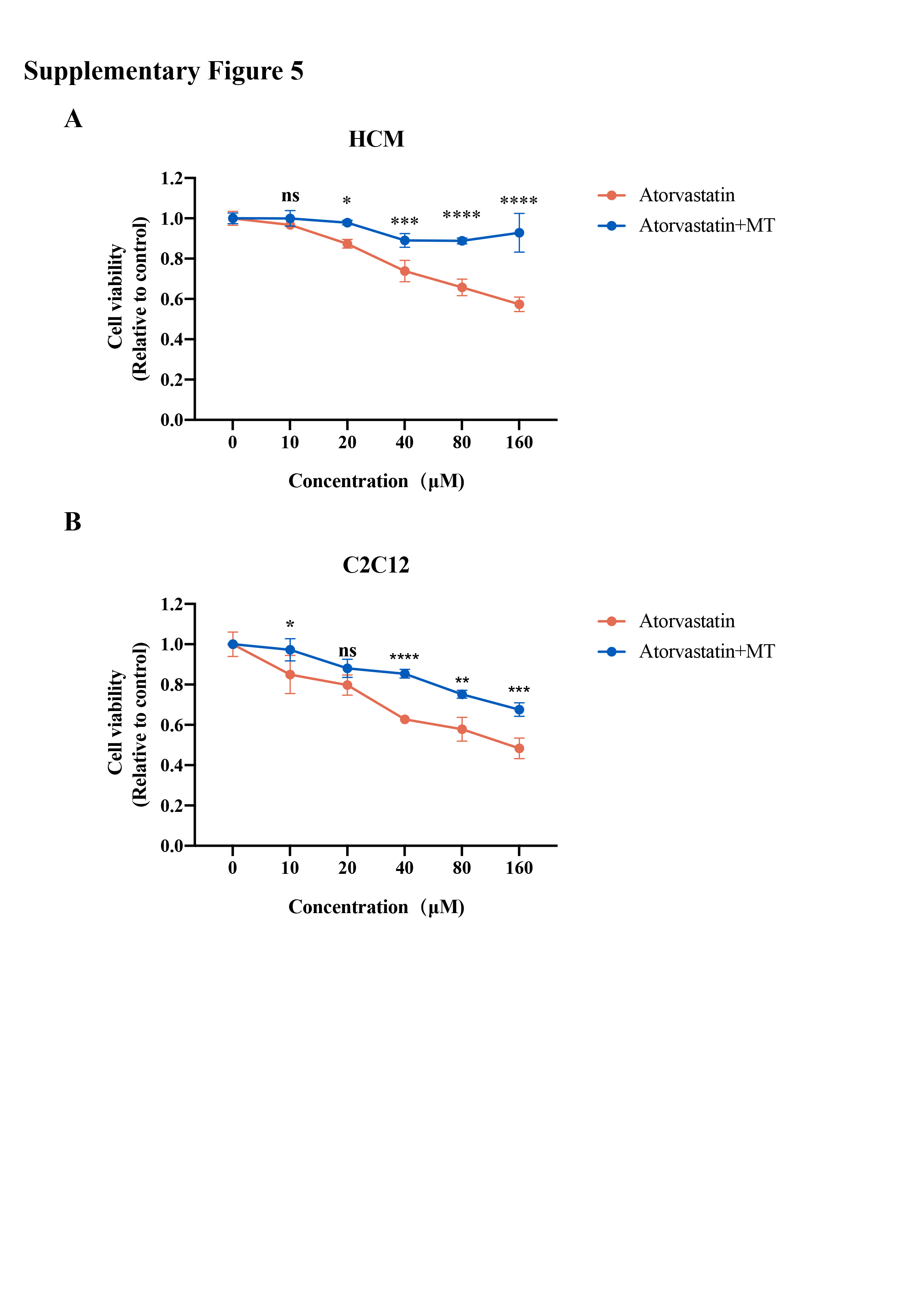

Supplement: Supplementary file 6 [file Image5.tif]
